# Use of a comprehensive systemic ultrasound evaluation in the diagnosis and analysis of acute lateral region ankle sprain

**DOI:** 10.1186/s12891-023-06642-0

**Published:** 2023-06-23

**Authors:** Jae Hwang Song, Jeong Jae Moon, Woo Jin Shin, Kwang Pyo Ko

**Affiliations:** 1 Department of Orthopedic Surgery, Konyang University Hospital, Daejeon, Republic of Korea; 2 Department of Orthopedic Surgery, Changwon Hospital, Korea Worker’s Compensation & Welfare Service, Gyeongnam, Republic of Korea; 3 Hanmaeum Orthopedic Clinic, Daejeon, Republic of Korea

**Keywords:** Ankle sprain, Ultrasound, Anterior talofibular ligament, Midtarsal joint, Syndesmotic ligament

## Abstract

**Background:**

For the diagnosis of acute lateral ankle sprain, many clinicians use ultrasound; they typically focus on the lateral ligament complex, which is the most common site of lesions in ankle sprain. However, this approach risks missing other foot and ankle lesions. The present study aimed to provide and analyze the results of a new ultrasound method of diagnosis for acute lateral ankle sprain which can thoroughly investigate overall lesions of the foot and ankle.

**Methods:**

Retrospective cross-sectional cohort study of 123 patients who underwent diagnostic ultrasound within 1 week of acute lateral ankle injury was performed. Causes of ankle sprain, incidence and severity of each ligament injury, location of anterior talofibular ligament (ATFL) injury, accompanying ligament injury, and occult fracture were analyzed.

**Results:**

Among the 102 cases of ATFL injuries, 60 (58.5%) had islolated ATFL injury, 28 (27.5%) had accompanying calcaneofibular ligament injury (CFL), and 14 (13.7%) had accompanying midtarsal or syndesmosis injury. ATFL injuries occurred on the fibula attachment in 48 (47.1%) cases, ligament mid-substance in 24 (23.5%) cases, and talus attachment in 30 (29.4%) cases. Among the 165 lesions from 123 cases, injuries of the fourth or fifth dorsal tarsometatarsal (12 cases, 7.3%), bifurcate (11 cases, 6.7%), and anterior tibiofibular (11 cases, 6.7%) ligaments were not rare.

**Conclusion:**

These findings suggest that an ultrasound examination involving investigation of the midtarsal joint and syndesmotic ligament, as well as the ATFL and CFL, is useful for comprehensive, systemic diagnosis of acute lateral ankle sprain.

## Introduction

Ankle sprains are common, constituting approximately25% of all sports injuries; approximately 85% of these are lateral ankle sprains caused by inversion, involving injuries of the lateral ligament complex [1]. Approximately 33% of patients with ankle sprains have a reinjury within 3 years; approximately 25% progress to chronic ankle instability [2]. A surgical procedure such as anatomical repair or reconstruction of the anterior talofibular ligament (ATFL) or calcaneofibular ligament (CFL) is required for treatment of chronic ankle instability [3]. Therefore, accurate diagnosis and proper initial treatment for acute ankle sprain are necessary to prevent such long-term complications.

For the diagnosis of lateral ankle sprains, physical examination and simple radiographic imaging are frequently used [4]. In general, when diagnosing an acute ankle sprain, the tender point is identified in accordance with the Ottawa ankle rules; the potential need for simple ankle and foot radiographic images is determined [5]. On this basis, careful observation is emphasized to avoid missing any occult fracture [5]. Although magnetic resonance imaging (MRI) is highly accurate for diagnosing lateral ankle sprains and computed tomography is useful for identifying occult or small avulsion fractures, applications of these methods are restricted because of their high cost, time consumption, and other limitations [6, 7].

Recently, multiple reports have described the value of ultrasound in the diagnosis of ankle ligament injuries [7–9]. Because ultrasound imaging is inexpensive, free of radiation, and permits easy detection of small changes in soft tissues, it has received increasing interest for the analysis of foot and ankle injuries [10]. Furthermore, because any lesion can be observed by real-time dynamic imaging, ultrasound enables accurate diagnosis of ligament injury, focusing on the tender point and any avulsion fracture adjacent to the ligament attachment site [11, 12]. With the accurate physical examination by palpating tender point, point-of-care ultrasound offers physicians an opportunity to better define anatomy and pathophysiology, thus enhancing the diagnostic capabilities of acute ankle sprain [13]. Hence, it is essential to diagnose acute ankle sprain with ultrasound under thorough physical examination. However, during ultrasound scanning, many clinicians focus on the lateral ligament complex (i.e., ATFL and CFL), which is the most common area with a lesion in ankle sprain; these non-systematic ultrasound examinations risk misdiagnosis or missed identification of other foot and ankle injuries involving midfoot and syndesmotic lesions. For accurate and complete diagnosis, routine ultrasound examination of a wider area is an appropriate approach. Thus far, there are limited studies and data concerning the standardization of foot and ankle ultrasound scan methods or pathological definitions for the diagnosis and analysis of acute lateral ankle sprain.

The present study aimed to provide and analyze the results of a comprehensive systematic ultrasound method of diagnosis for acute lateral ankle sprain which thoroughly investigates overall lesions of the foot and ankle.

## Materials and methods

### Participants and study design

The protocol for this cross-sectional cohort study was approved by our institutional review board (IRB number protocol number: KYUH 2021–12-016). Among the patients who underwent ultrasound examination within 1 week after lateral region ankle injury between January and December 2018, 199 patients with acute ligament injury or fracture were initially enrolled in the study. Patients with ankle injuries underwent ankle anteroposterior, lateral, and mortise X-rays; patients with foot injuries underwent foot anteroposterior, lateral, and oblique X-rays. All patients included in the study underwent both ultrasonography and radiography examinations. The inclusion criteria for participants were an alert and stable condition; absence of penetrating trauma, open fractures, dislocations, major trauma, and osteomyelitis; and positive Ottawa ankle rules [5]. The exclusion criteria were ankles with a definite fracture without ligament injury; previous ankle surgery (ligament repair or internal fixation of metal implant); ankle deformity (cavus foot, flatfoot, or hindfoot malalignment); and direct injury, including traffic accidents. Finally, 76 patients were excluded from the analyses, resulting in a final cohort of 123 patients (123 ankles). Along with the ultrasonography and radiography findings, we analysed the causes of ankle sprain according to sex and age group, incidence and severity of each ligament injury, location of ATFL injury, accompanying ligament injury, and comparison of occult fracture detection between simple radiography and ultrasonography. The cause of each acute lateral ankle injury was classified as walking on even surfaces, walking on uneven surfaces such as stairs, or sports injury including running.

### Ultrasound examination

**Fig. 1:**
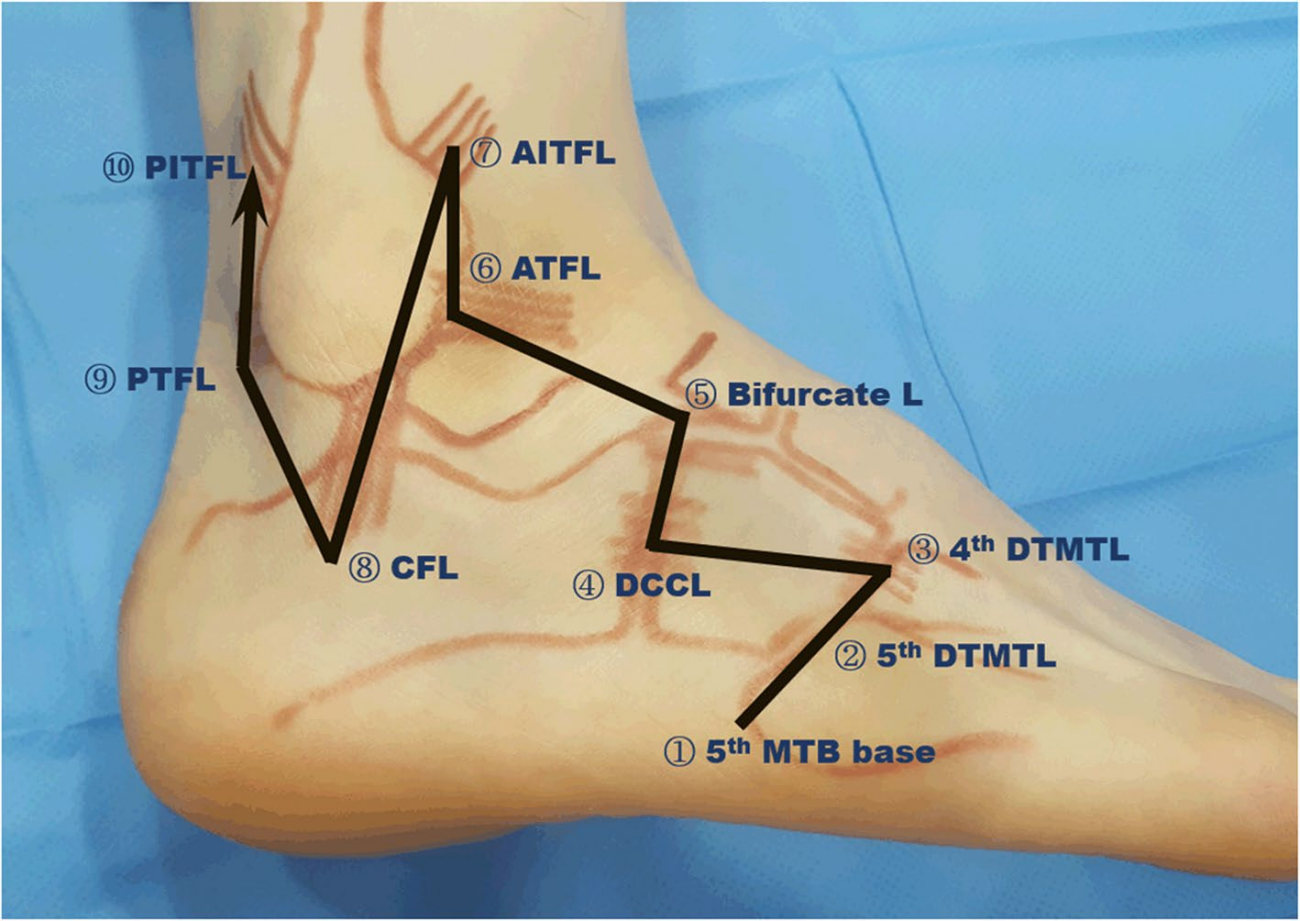
Surface anatomy and ultrasound examination of ankle. Ultrasound examination was performed in the order of the indicated numbers. 5th MTB, fifth metatarsal bone; 5^th^ and 4^th^ DTMTLs, fifth and fourth dorsal tarsometatarsal ligaments; DCCL, dorsal calcaneocuboid ligament; L, ligament; ATFL, anterior talofibular ligament; AITFL, anterior inferior tibiofibular ligament; CFL, calcaneofibular ligament; PTFL, posterior talofibular ligament; PITFL, posterior inferior tibiofibular ligament

An ultrasound examination was performed with a linear probe (8 − 17 MHz) using one instrument (E-CUBE Platinum; ALPINION Medical Systems, Seoul, South Korea). Each patient lay in the supine position with one leg supported by a pillow. Prior to the ultrasound examination, the patient was instructed to relax their muscles. The patient maintained plantar flexion and inversion from the ankle in the supine position [9]. Beginning at the base of the fifth metatarsal bone, where the tendon of the peroneus brevis muscle is attached, we observed the fourth and fifth dorsal tarsometatarsal ligaments (4^th^ & 5^th^ DTMTLs), dorsal calcaneocuboid ligament, and bifurcate ligament (Fig. 1). The bifurcate ligament consists of calcaneocuboid ligament laterally and calcaneonavicular ligament medially. To identify the bifurcate ligament, the calcaneocuboid ligament (lateral band) was identified as a band from the anterior process of the calcaneus to the cuboid parallel to the dorsal calcaneocuboid ligament; the calcaneonavicular ligament (medial band) was identified by slightly shifting the distal portion of the probe towards the navicular bone.

**Fig. 2:**
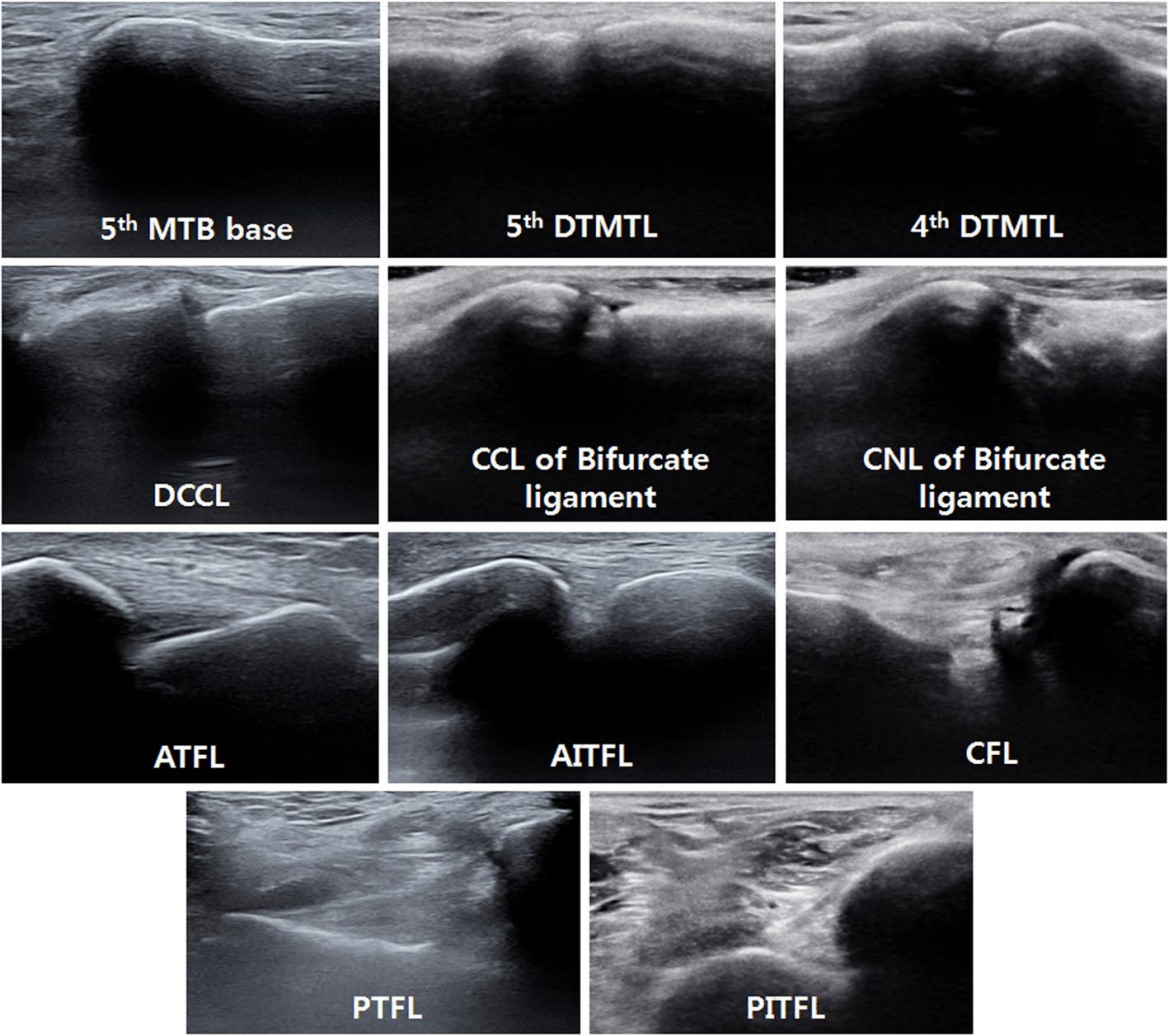
Normal ultrasound findings of each ligament. 5th MTB, fifth metatarsal bone; 5^th^ and 4^th^ DTMTLs, fifth and fourth dorsal tarsometatarsal ligaments; DCCL, dorsal calcaneocuboid ligament; CCL, calcaneocuboid ligament; CNL, calcaneonavicular ligament; ATFL, anterior talofibular ligament; AITFL, anterior inferior tibiofibular ligament; CFL, calcaneofibular ligament; PTFL, posterior talofibular ligament; PITFL, posterior inferior tibiofibular ligament

The transducer was sequentially placed over the bony landmarks of the ATFL, consistent with the position described by Matsui et al. [14]. The origin of the ATFL was detectable immediately anterosuperior to the fibular obscure tubercle; the insertion of the ATFL was located 60% of the distance from the inferolateral corner to the anterolateral corner of the talar body along the anterior border of the talar body–neck junction [14]. The ATFL was examined in resting and stress positions; the stress position was achieved by manual application of plantar flexion and inversion stress by the examiner [9]. The ultrasound probe was positioned parallel to the sole of the foot for identification of the linear fibril pattern of the ATFL. The anterior inferior tibiofibular ligament (AITFL) was examined between the anterior fibular tubercle and the anterior tubercle of the distal tibia while the ankle was under dorsiflexion and external rotation [15]. Then, the origin of the CFL was detectable immediately posteroinferior to the fibular obscure tubercle; the insertion of the CFL was examined posterosuperior to the peroneal tubercle [14]. The CFL was examined while the ankle was under dorsiflexion and inversion. The CFL lies between the calcaneus and the peroneal tendons; it forms a hammock and fibrous pulley for the tendons. Hence, if the ankle is dorsiflexed and inverted, the intact CFL will be stretched, causing superficial migration of the peroneal tendons [16]. Subsequently, the posterior talofibular ligament and posterior inferior tibiofibular ligament were examined while the ankle was under dorsiflexion. The posterior talofibular ligament was identified between the posterior aspect of the distal fibula and the lateral tubercle of the talar posterior process. The posterior inferior tibiofibular ligament was identified between the posterolateral tibial tubercle and the posterior fibular tubercle (Fig. 2).

### Interpretation of ultrasound findings

**Fig. 3:**
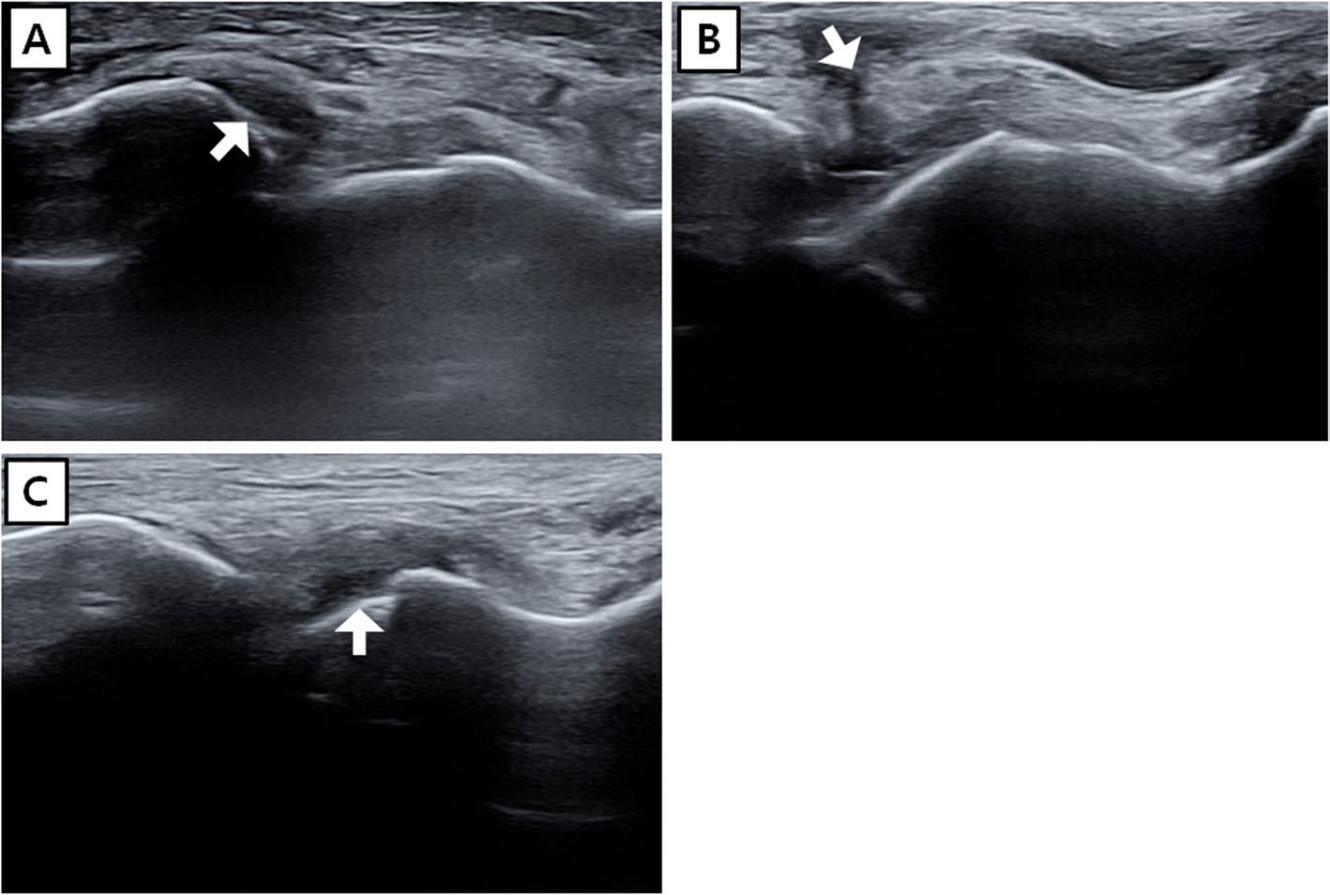
Location of anterior talofibular ligament injury: **A** fibula attachment, (**B**) mid-substance, and (**C**) talus attachment. White arrows indicate lesions

Ligament injury was classified as ligament substance injury (including incomplete and complete tears) or avulsion fracture. An incomplete tear was defined as a diffuse hypoechoic lesion with ligament swelling [17]. A complete tear was defined as a hypoechoic discontinuity, accompanied by wavy, free ligament ends with hematoma around the lesion [17]. A complete tear was also identified on the basis of dynamic ultrasound findings that included a > 2 mm change in distance between the origin and insertion of the ligament, compared to the healthy side, because of external force. Injury to the ATFL, the ligament most commonly injured in lateral ankle sprain, was specified by division of the ATFL into three parts: the fibula attachment, mid-substance, and the talus attachment (Fig. 3). Ultrasound examinations were performed and interpreted by two skilled orthopaedic surgeons who had more than 10 years of experience in musculoskeletal ultrasound examination.

### Statistical analysis

The outcomes of the present cross-sectional cohort study were statistically analyzed. For statistical analysis, SPSS Statistics software (version 22; IBM Co.; Armonk, NY, USA) was used. The chi-squared test or Fisher’s exact test was performed for analysis of categorical data. *P*-values < 0.05 were considered statistically significant.

## Results

A final cohort of 123 patients was eligible for this study (mean age, 32.8 years). There were 50 men (mean age, 31.7 years); and 73 women (mean age, 33.5 years); 32 were aged 10–19, 30 were in their 20 s, 18 were in their 30 s, 21 were in their 40 s, 16 were in their 50 s, and 6 were in their 60 s or older.

### Causes of injury according to sex and age group

The causes of ankle injury according to sex and age group are listed in Table 1. Fourteen (28% of men), 20 (40% of men), and 16 (32% of men) men experienced injuries when walking on even surfaces, walking on uneven surfaces, and playing sports, respectively. Twenty-seven (37% of women), 35 (48% of women), and 11 (15% of women) women experienced injuries when walking on even surfaces, walking on uneven surfaces, and playing sports, respectively.

**Table 1:** Causes of injury according to sex and age group

Sex	Cause	Age						Cases
		< 20	20–29	30–39	40–49	50–59	≥ 60	
Male	Even surface ambulation	3	3	0	4	2	2	14
	Uneven surface ambulation	5	4	2	6	2	1	20
	Sports	9	3	2	2	0	0	16
	Total	17	10	4	12	4	3	50
Female	Even surface ambulation	3	9	1	6	7	1	27
	Uneven surface ambulation	7	10	11	2	3	2	35
	Sports	5	1	2	1	2	0	11
	Total	15	20	14	9	12	3	73
Total		32	30	18	21	16	6	123

Values are expressed as n. (the number of cases)

### Incidence and severity of injury to each ligament

Ligament substance injuries (including incomplete and complete tears) and avulsion fractures were found in 165 lesions in 123 patients. The ATFL was the most common lesion site, found in 102 (61.8%) cases, followed by the CFL (29 cases, 17.5%), 4^th^ & 5^th^ DTMTLs (12 cases, 7.3%), bifurcate ligament (11 cases, 6.7%), and AITFL (11 cases, 6.7%). Among the ligament injuries, there were 66 cases of incomplete tear (40.0%), 63 cases of complete tear (38.2%), and 36 cases of avulsion fracture (21.8%). All 4^th^ & 5^th^ DTMTL injuries were avulsion fractures (Table 2).

**Table 2:** Incidence and severity of injury to each ligament

	4 ~ 5^th^ DTMTLs	DCCL	Bifurcate L	ATFL	AITFL	CFL	PTFL	PITFL	Cases
Incomplete tear	0	0	0	46	4	16	0	0	66 (40.0)
Complete tear	0	0	0	46	6	11	0	0	63 (38.2)
Avulsion Fx	12	0	11	10	1	2	0	0	36 (21.8)
Total	12 (7.3)	0	11 (6.7)	102 (61.8)	11 (6.7)	29 (17.5)	0	0	165 (100)

Values are expressed as n (%). *4 ~ 5*^*th*^* DTMTLs* Fourth and fifth dorsal tarsometatarsal ligaments, *DCCL* dorsal calcaneocuboid ligament, *L* Ligament, *ATFL* Anterior talofibular ligament, *AITFL* Anterior inferior tibiofibular ligament, *CFL* Calcaneofibular ligament, *PTFL* Posterior talofibular ligament, *PITFL* Posterior inferior tibiofibular ligament

### Patients with more than one ligament injury

There were 84 (68.3%) patients with a single injury, 36 (29.3%) patients with double injuries, and 3 (2.4%) patients with triple injuries. All 39 patients with complex ligament injuries showed an injury of the ATFL. In addition, all patients with injuries including more than two sites had accompanying ATFL and CFL injuries. Of the 29 cases with a CFL injury, 28 cases (96.6%) were accompanied by an ATFL injury (Table 3).

**Table 3:** Accompanying ligament injury

Injury	Involvement	Cases (*n* = 123)
Single		84 (68.3)
Double		36 (29.3)
	ATFL–CFL	25
	ATFL–AITFL	6
	ATFL–Bifurcate L	2
	ATFL–4^th^ & 5^th^ DTMTLs	3
Triple		3 (2.4)
	ATFL–CFL–AITFL	2
	ATFL–CFL–Bifurcate L	1

Values are expressed as n (%). 4^th^ & 5^th^ DTMTLs, fourth and fifth dorsal tarsometatarsal ligaments, *L* ligament, *ATFL* Anterior talofibular ligament, *AITFL* Anterior inferior tibiofibular ligament, *CFL* calcaneofibular ligament

### Location of ATFL injury

ATFL injuries occurred at the fibula attachment in 48 (47.1%) cases, ligament mid-substance in 24 (23.5%) cases, and talus attachment in 30 (29.4%) cases. Among the 102 cases of ATFL injuries, 60 (58.5%) had only ATFL injury, 28 (27.5%) had an accompanying CFL injury, and 14 (13.7%) had an accompanying midtarsal or syndesmosis injury. There was no significant difference between the location of ATFL injury and the presence of CFL injury (*P* = 0.197) or AITFL injury (*P* = 0.144; Table 4).

**Table 4:** Location of anterior talofibular ligament (ATFL) injury

		Isolated ATFL	CFL	AITFL	Bifurcate L	4^th^ & 5^th^ DTMTLs	Cases
ATFL injury	Fibula attachment	28	12	5	2	1	48 (47.1)
	Ligament mid- substance	16	7	1	0	0	24 (23.5)
	Talus attachment	16	9	2	1	2	30 (29.4)
Total		60	28	8	3	3	102

Values are expressed as n (%). CFL, calcaneofibular ligament; *AITFL* Anterior inferior tibiofibular ligament, *L* ligament; *4*^*th*^* & 5*^*th*^* DTMTLs* Fourth and fifth dorsal tarsometatarsal ligaments

### Comparison of occult fracture detection between plain X-ray and ultrasound

**Fig. 4:**
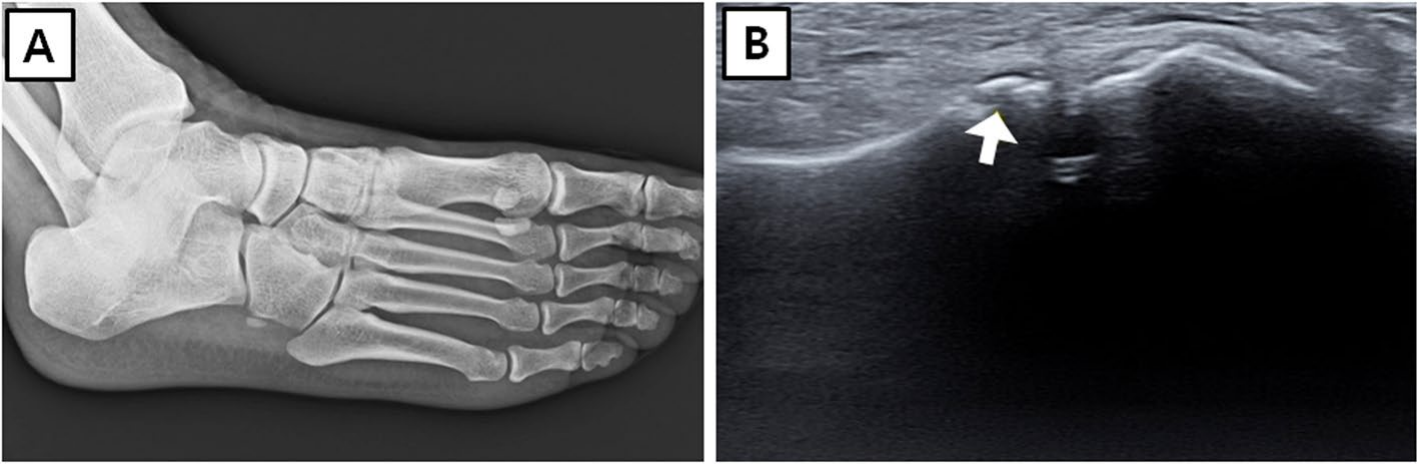
Occult fracture detection by ultrasound. A 44-year-old woman experienced an inversion sprain of right ankle. Fracture was not observed in (**A**) a plain X-ray, but cuboid fracture of the fourth tarsometatarsal joint was identified in (**B**) an ultrasound image. White arrow indicates fracture line

Among the 123 cases, there were 35 cases (28.5%) of fractures observed in both ultrasound images and plain X-rays. Fracture was found in neither ultrasound images nor plain X-rays in 86 cases (69.9%). There were 2 cases (1.6%) where fractures were observed in ultrasound images but not in plain X-rays (Fig. 4).

## Discussion

The most important findings of the present study are that the cases of lateral region ankle sprain which exhibited the midtarsal joint and syndesmotic ligament, as well as the ATFL and CFL, were not unusual. These findings were demonstrated by the comprehensive systematic ultrasound method which thoroughly investigated overall lesions of the foot and ankle.

Based on insurance data in the United States from 2007 to 2011, Feger et al. [4] reported that lateral ankle sprains constituted 96.2% of all ankle sprains; the incidence was significantly higher in women than in men. Furthermore, the proportion of patients under 20 years old was highest, at 35.7%; the incidence rate decreased with age [4]. Waterman et al. [12] reported that sports injuries led to high incidences of lateral ankle sprains in men under 20 years old and in women over 30 years old. In the present study, women had more ankle injuries than men had (Table 1). The incidence was highest in patients under 20 years old, and the most common cause in those ages was sports injury. There was a tendency toward fewer sports-related lateral ankle injuries with increasing age.

MRI and ultrasonography are commonly used to evaluate the ligament injuries of ankle [7, 8, 18]. However, MRI has some limitations for the diagnosis of ankle instability for several reasons [7]. First, it does not allow dynamic imaging; thus, it is unable to gauge the instability of the affected ankle. Second, the usefulness of an MRI image heavily depends on the cut precision. Third, MRI is expensive and time-consuming. Recently, many reports have described the value of ultrasound in the diagnosis of ankle ligament pathology [9–11, 19]. Ultrasound is easily available, economical, and portable. Because of device and technique developments, ultrasound shows high sensitivity and specificity, comparable to MRI; it also has the advantage of dynamic imaging [8, 10, 20]. Furthermore, it permits the visualisation of a large number of joints in a short period, along with comparative investigation of the contralateral limb [10]. In the present study, we used systematic evaluation for diagnosis of acute lateral ankle and foot ligamentous injuries; it can investigate overall lateral lesions on the foot and ankle in detail, including the midtarsal joint and syndesmotic ligament as well as the lateral ligament complex (Fig. 1). Our method consists of standardized probe scanning which can simultaneously investigate multiple meaningful lesions of lateral ankle sprain.

Previous studies [21] have reported that the ATFL is the most commonly damaged ligament in acute lateral ankle injury. In the present study, 102 cases (82.9%) of ATFL injury were identified among 123 cases, a result that is consistent with previous data (Table 2). The maximum load to failure of the CFL is 2.5 − 3.5-fold greater than maximum load to failure of the ATFL; the CFL experiences injury during dorsiflexion and adduction of the ankle [22]. The CFL was the second most commonly injured ligament in this study cohort (29 cases, 24.2%); 28 (96.6%) of the 29 cases of CFL injury were accompanied by ATFL injury (Table 3). Broström [23] also found combined ruptures of the ATFL and CFL in 20% of cases; Isolated rupture of the CFL was very rare. During lateral ankle injuries, the ATFL is damaged by excessive plantar flexion and inversion; the talus is then stabilised by the mortise. However, we presume that CFL injury will be caused by additional inversion of the talus and adduction of the calcaneus via subsequent transmission of external force.

Previous study reported that injury of the ATFL mid-substance is common, whereas injury of the ATFL talus attachment is rare [24]. This is because the site of the talus attachment of the ATFL has a higher bone density than the fibula attachment, along with a wider attachment area around the talus body–neck junction [25]. It is common to observe injury of the ATFL mid-substance in the presence of remnant ligament parenchyma in the fibula and talus attachment [26]; we classified the ligament parenchyma into three parts in the present study (Fig. 3). In the present study, injury of the ATFL mid-substance was less common, at 24 cases, while injury of the ATFL talus attachment was relatively common, at 30 cases; these findings differ from the results in previous studies (Table 4) [24, 25]. Our findings suggest that careful evaluation is necessary for the fibula and talus attachment site of the ATFL, as well as mid-substance.

When ligament lesions are assessed after acute ankle sprain, attention is typically directed at the ATFL and the CFL [27]. Lesions to the midtarsal joints have primarily been noted in cases of high-velocity trauma. However, Broström [28] noticed that 20% of patients with acute ankle sprains had clinical signs of injury to the bifurcate or dorsal calcaneocuboid ligaments; those lesions were regarded as common injuries, and identification of the midtarsal injury was considered important because its recovery may be protracted without proper treatment. Another study [29] reported that 24.5% of acute ankle injuries showed evidence of an acute syndesmosis injury. Hence, we investigated the midtarsal joint and syndesmotic ligament, as well as the lateral ligament complex. In the present study, among the 165 lesions from 123 cases, injuries of the 4^th^ & 5^th^ DTMTLs (12 cases, 7.3%), bifurcate ligament (11 cases, 6.7%), and AITFL (11 cases, 6.7%) were not unusual (Table 2). Moreover, among the 102 cases of ATFL injuries, 60 (58.5%) had only ATFL injury, 28 (27.5%) had accompanying CFL injury, and 14 (13.7%) had accompanying midtarsal or syndesmosis injury (Table 4). These findings suggest a need for ultrasound examination which investigates the midtarsal joint and syndesmotic ligament, as well as the ATFL and CFL.

Our study found 2 cases in which no avulsion fracture was visible on a simple radiograph but was diagnosed on ultrasound (Fig. 4). Avulsion fractures typically occur in occult fractures, which are difficult to diagnose with simple radiographic images; thus, careful physical examination has been emphasized in accordance with the Ottawa ankle rules [30]. Sali et al. [10] reported that ultrasound sensitivity in detecting fractures was 100% (95% confidence interval, 83.8 − 100%) and its specificity was 99.1% (95% confidence interval, 95 − 99.8%). Simanovsky et al. [31] emphasized the superior sensitivity and specificity of ultrasound, compared to simple radiographs, for occult fractures of ankles in paediatric patients [31]. Avulsion fractures accompanied 21.8% of the lateral ankle injuries in our study. Depending on the nature of injury (i.e., pure ligament injury or avulsion fracture), the period of treatment varies, including the duration of splint application and rehabilitation. If patients do not undergo proper initial treatment because of misdiagnosis, they are more likely to develop chronic complications [32]. Hence, there is a need for thorough investigation of avulsion fracture by ultrasound.

To our knowledge, this is the first retrospective cross-sectional cohort study concerning the ultrasound method composed of standardized probe scanning which can simultaneously investigate the midtarsal joint and syndesmotic ligament, as well as the lateral ligament complex—and its results for the diagnosis of acute lateral ankle sprain. We have proposed the proper foot and ankle position, and probe position for ultrasound examination of each ligament. The present study also investigated the frequencies of accompanying acute ligament injuries and avulsion fractures, as well as the severity and location of injury of the ATFL within 1 week of lateral ankle injury.

The present study had several limitations. First, it lacked external validity because of the small study population. Therefore, our data regarding the incidence of ligament injuries in acute ankle sprain cannot be generalized to the whole population. Second, this was a retrospective cohort study; thus, the accuracy of demographic data involving patients with acute ankle sprain cannot be guaranteed. Third, the study did not investigate the results of follow-up treatment or progression to lateral ankle instability. To complement the limitations of this study, additional prospective studies are needed that involve long-term treatment assessments in large numbers of patients. Notably, the ultrasound examination described in the present study may be difficult for an unskilled or untrained orthopaedic surgeon. However, we believe that our ultrasound examination protocol can be helpfully and easily used by clinicians who have more than 2 − 3 years of clinical experience with ultrasound imaging.

In conclusion, the findings in this study suggest that an ultrasound examination which involves investigation of the midtarsal joint and syndesmotic ligament, as well as the ATFL and CFL, is useful for accurate diagnosis of acute lateral ankle sprain. By using the proposed, standardized, ultrasound examination method and providing adequate initial treatment, clinicians can prevent the onset of chronic complications in patients with acute lateral ankle injury.

## Data Availability

The data that support the findings of this study are available from the corresponding author upon reasonable request**.**
